# Is Accessibility to Dental Care Facilities in Rural Areas Associated with Number of Teeth in Elderly Residents?

**DOI:** 10.3390/ijerph14030327

**Published:** 2017-03-21

**Authors:** Tsuyoshi Hamano, Miwako Takeda, Kazumichi Tominaga, Kristina Sundquist, Toru Nabika

**Affiliations:** 1 Institute of General Education, Kyoto Sangyo University, Motoyama, Kamigamo, Kita-ku, Kyoto 603-8555, Japan; 2 Department of Functional Pathology, Shimane University School of Medicine, 89-1 Enya-cho, Izumo, Shimane 693-8501, Japan; nabika@med.shimane-u.ac.jp; 3 Center for Community-Based Health Research and Education (CoHRE), Organization for the Promotion of Project Research, Shimane University, 223-8 Enya-cho, Izumo, Shimane 693-8501, Japan; 4 Tominaga Dental Office, 97-3 Yamada, Ohnan-cho, Ohchi, Shimane 696-0313, Japan; shika-t@ohtv.ne.jp; 5 Center for Primary Health Care Research, Lund University, Clinical Research Centre (CRC), Building 28, Floor 11, Jan Waldenströms Gata 35, Skåne University Hospital, SE-205 02 Malmö, Sweden; kristina.sundquist@med.lu.se; 6 Stanford Prevention Research Center, Stanford University, Medical School Office Building (MSOB), 251 Campus Drive MC 5411, Stanford, CA 94305, USA

**Keywords:** dental care accessibility, number of teeth, rural area, cross-sectional study

## Abstract

Given that public transportation networks are less developed in rural than in urban areas, a lack of accessibility to dental care facilities could be a barrier to routine dental checkups. Thus, we hypothesized that the distance to the dental care facilities is a risk factor for tooth loss. The aim of this study was to test whether there is an association between the distance to dental care facilities, estimated by geographic information systems, and number of teeth, assessed by an oral examination, among elderly residents of a rural area in Japan. Data were collected in 2016 from a cross-sectional study conducted in Shimane prefecture, Japan. After excluding participants with missing data (*n* = 21), we analyzed data from 710 participants. Of them, 40.6% were male and the mean (standard deviation) age was 67.4 (7.4) years. Further, 68.0% (*n* = 483) had at least 20 teeth. We found that the distance to dental care facilities was significantly associated with the number of teeth (less than 20) (odds ratio = 1.07, 95% confidence interval = 1.01–1.12) after adjustment for potential confounders. This result suggested that individuals without easy access to dental care facilities may be important targets for dental care.

## 1. Introduction

Previous studies have indicated that tooth loss in elderly populations is associated with mortality [1,2,3]. Japan is an aging society and the percentage of the elderly in 2060 will reach 39.9% [4]. Maintaining dental health in an aging population is a major public health concern in Japan.

Several risk factors affecting tooth loss have been suggested, including sociodemographic factors (e.g., age and educational attainment) [5,6], lifestyle and health factors (e.g., nutritional status, smoking habits, and current history of disease) [5,6,7,8,9], and oral health conditions and behaviors (e.g., complaint of oral condition and dental care visits) [10,11]. Distance to dental care facilities is also a potential factor for tooth loss, especially in rural areas [12]. Given that public transportation networks are often less developed in rural compared to urban areas, a lack of accessibility to dental care facilities could be a barrier to routine dental checkups. We hypothesized that the distance to dental care facilities is a risk factor for tooth loss, assessed by an oral examination. To the best of our knowledge, no previous studies have examined this association in a rural area of Japan.

In Japan, a list of targets for Health Japan 21 (the second term) was published in 2012 [13]. This is a 10-year plan that began in 2013 and the government will work toward increasing the number of people with 20 teeth at the age of 80 years (8020 campaign) [13]. The aim of this cross-sectional study was to test whether there is an association between the distance to dental care facilities, estimated by geographic information systems (GIS), and the number of teeth (less than 20) among elderly residents in a rural area.

## 2. Materials and Methods

### 2.1. Study Design

Data was gathered from participants in the Shimane Center for Community-Based Health Research and Education (CoHRE) Study. The Shimane CoHRE study is a cohort study to examine the determinants of lifestyle-related diseases, including oral health, in rural areas in the southern part of Shimane prefecture, Japan [14,15,16,17,18]. The present study was conducted as a cross-sectional study of Shimane CoHRE study for which participants were recruited in 2016. 731 of the residents who were covered by the National Health Insurance and aged between 40 and 74 years of age in the town of Ohnan participated in the 2016 survey. After excluding participants with missing data (21 participants, 2.9%), we analyzed data from 710 participants. The Ethics Committee of the Shimane University School of Medicine approved the study protocol in 2016 (number 2227, 5/12). Written informed consent was obtained from all participants.

### 2.2. Number of Teeth

Dental examination was conducted by a trained dental hygienist, with both the dental hygienist and participants in a seated position. The number of teeth was counted in the examination, and participants were divided into the following two categories: those with less than 20 teeth and with 20 or more teeth [13].

### 2.3. Distance to Dental Care Facilities

The Geographic Information Systems software (ArcGIS, version 10.0, Environmental Systems Research Institute, Redlands, CA, USA) was employed for database queries and used to estimate distance to dental care facilities from the individuals’ addresses. Network analysis, which determined the shortest path between the participant locations and the dental care facilities, was performed on road networks.

### 2.4. Other Measures

Age (years, analyzed as a continuous variable), gender (male vs. female), body mass index (BMI) (analyzed as a continuous variable), current smoker (yes vs. no), current alcohol drinker (yes vs. no), with regular physical activity (engaged in regular physical activity = yes vs. not engaged in regular physical activity = no), medication for disease treatment (medication against hypertension, diabetes mellitus and hyperlipidemia, yes vs. no), receiving a dental health check within the past one year (yes vs. no), having any oral health problems (yes vs. no), having enough sleep (yes vs. no), elevation estimated by the GIS (median value, ≤258 m vs. >258 m), and accessible transportation (driver = yes vs. non-driver = no). Accessible transportation was assessed by the following question: “Do you have a valid driving license and regularly drive a car?” (if yes = driver, if no = non-driver).

### 2.5. Statistical Analysis

The χ^2^ and *t*-tests were used to compare characteristics according to the number of teeth (less than 20 vs. 20 or more). Multivariable logistic regression model was performed to derive odds ratios (ORs), 95% confidence intervals (95% CIs), and *p*-values. *p*-values less than 0.05 were considered statistically significant. All statistical analyses were performed using IBM SPSS Statistics 20 (IBM Corporation, Tokyo, Japan).

## 3. Results

The characteristics of the study participants are shown in Table 1 by number of teeth. There were statistically significant differences between the two groups (less than 20 teeth vs. 20 teeth or more) in the distance to dental care facilities, age, gender, medication (hypertension), and car driver. On the other hand, there were no statistically significant differences in current smoker, current alcohol drinker, regular physical activity, medication (diabetes mellitus and hyperlipidemia), BMI, elevation, having enough sleep, use of dental health checks, and having any oral health problems.

Table 2 shows the results of the multivariable logistic regression analysis. The distance to dental care facilities was significantly associated with the number of teeth (less than 20) (OR = 1.07 per km, 95% CI = 1.01–1.12). The factors of age, non-smoker, and non-driver were also significantly associated with the number of teeth (less than 20) (OR = 1.19, 95% CI = 1.14–1.25, OR = 0.47, 95% CI = 0.25–0.90, and OR = 1.88, 95% CI = 1.12–3.13, respectively).

## 4. Discussion

Although a previous study conducted in a rural region of the United States examined the association between the distance to care facilities and care visits [12], no studies have been performed on the potential effects of distance to dental care facilities, estimated by GIS, on the number of teeth, assessed by an oral examination, in a rural area of Japan. Our results showed that the distance to dental care facilities increased the OR of the number of teeth (less than 20) (OR = 1.07, 95% CI = 1.01–1.12), independently of sociodemographic factors, lifestyle, and oral health conditions. 

The present result is consistent with our previous study, indicating that residential location influences health conditions; a cross-sectional study conducted in a rural area found that distance from a city center affected the incidence of hypertension [17]. Generally, health care facilities in a rural area are clustered at the center of the town. Although the average distance to dental care facilities was 3.8 km for the participants in this study, this may be too far for the elderly residents. Thus, further studies are required to examine the burden of accessibility to dental care facilities by distance.

A previous study pointed out that transportation available for residents should be considered when discussing the association between distance to health care facilities and residents’ health [12]. Although information about modes of transportation to dentists (e.g., use of public transportation or of a ride offered by neighbors or family members) was not available in this study, we included the driving status of participants which might be an important determinant of a moving range in a rural area [12]. In our analysis, the distance to dental care facilities was associated with the number of teeth, independently of the driving status. We also tested the logistic regression model without a driver. As a result, the OR for the distance to dental care facilities was a similar value (OR = 1.06, 95% CI = 1.01–1.06, *p* = 0.016). Although more research is needed to examine reasons for why similar associations were shown, these results implicated that another explanatory factor (e.g., socio-economic status) should be considered to explain the association between accessibility to dental care facilities and the number of teeth. For example, previous studies revealed that lower socio-economic status is associated with the utilization of dental care and oral health behavior [19,20]. In addition, locational differences in attitude about dental care may account for the residential variation in the use of dental care [21].

The present study has several strengths. To our knowledge, this is the first study to examine the potential association between the distance to dental care facilities, estimated by GIS, and the number of teeth, assessed by an oral examination, in a rural area of Japan. GIS is a computer-based system that integrates and analyzes spatial data, including latitude and longitude, and its application to epidemiological research has been increasing in recent years [22]. This approach contains less measurement noise than the information evaluated by respondents’ responses to questions as to the distances, because GIS can estimate the distance between population locations and health care facilities based on road network information. Furthermore, the number of teeth was counted by dental hygienists rather than relying on self-reported data. On the other hand, there are also a number of potential limitations in the current study. First, due to the cross-sectional study design, it is difficult to argue the causal relationship between independent and dependent parameters. Second, our results could be explained by other unmeasured risk factors for tooth loss (e.g., socio-economic status and dietary intake). This is particularly evident in socioeconomic factors. Previous studies revealed that a low education level was associated with tooth loss [6,23]. The reason is that education level is associated with the utilization of dental care and oral health behavior [19,20]. Our data could not evaluate differences in socio-economic status between the center of the town and remote areas, so further studies are required to examine this issue. Third, our data did not include accurate information on years of smoking and volume of alcohol intake. Fourth, our analyses could not consider the effect of residence year. Finally, misclassification may have occurred in the self-reported data as a consequence of recall errors. 

## 5. Conclusions

The distance to dental care facilities was a significant risk factor for tooth loss, independently of sociodemographic, lifestyle and behavioral factors. Those who reside in a location far from dental care facilities may be important targets for dental care.

## Figures and Tables

**Table 1:** Characteristics of study participants.

	Number of Teeth (Less than 20) (*N* = 227)		Number of Teeth (20 or More) (*N* = 483)		*p*-Value
	*n*	% or Mean (SD)	*n*	% or Mean (SD)	
Distance to dental care facilities (per 1 km)	227	4.3 (3.6)	483	3.5 (3.3)	0.005
Age (per one year)	227	70.9 (3.7)	483	65.8 (8.1)	<0.001
Gender (male vs. female)	106	46.7	182	37.7	0.023
Current smoker (yes vs. no)	28	12.3	41	8.5	0.107
Current alcohol drinker (yes vs. no)	122	53.7	243	50.3	0.393
With regular physical activity (yes vs. no)	117	51.5	229	47.4	0.305
Medication (yes vs. no)					
Hypertension	89	39.2	145	30.0	0.015
Diabetes mellitus	32	14.1	50	10.4	0.145
Hyperlipidemia	65	28.6	127	26.3	0.513
Body Mass Index (per 1 kg/m^2^)	227	23.1 (3.2)	483	22.7 (3.4)	0.158
Driver (yes vs. no)	184	81.1	429	88.8	0.005
Elevation (≤258 m vs. >258 m)	103	45.4	250	51.8	0.113
Having enough sleep (yes vs. no)	163	71.8	338	70.0	0.618
Use of dental health checks (no vs. yes)	112	49.3	205	42.4	0.085
Having any oral health problems (no vs. yes)	158	69.6	321	66.5	0.404

SD, standard deviation.

**Table 2:** Multivariable logistic regression analysis with the number of teeth as the dependent variable.

Variables	OR	95% CI	*p*-Value
Distance to dental care facilities (per 1 km)	1.07	1.01–1.12	0.009
Age (per 1 year)	1.19	1.14–1.25	<0.001
Gender (male vs. female)	0.76	0.50–1.17	0.220
Current smoker (yes vs. no)	0.47	0.25–0.90	0.023
Current alcohol drinker (yes vs. no)	1.01	0.69–1.47	0.948
With regular physical activity (yes vs. no)	0.90	0.63–1.29	0.595
Medication (yes vs. no)			
Hypertension	0.97	0.66–1.42	0.889
Diabetes mellitus	0.96	0.56–1.63	0.886
Hyperlipidemia	1.06	0.71–1.58	0.764
Body Mass Index (per 1 kg/m^2^)	1.04	0.99–1.10	0.111
Driver (yes vs. no)	1.88	1.12–3.13	0.016
Elevation (≤258 m vs. >258 m)	1.26	0.87–1.80	0.210
Having enough sleep (yes vs. no)	0.94	0.63–1.39	0.773
Use of dental health checks (no vs. yes)	0.70	0.49–1.00	0.051
Having any oral health problems (no vs. yes)	1.02	0.70–1.50	0.890

Independent variables were coded as follows: gender (0 = male, 1 = female), current smoker, current alcohol drinker, medication for disease treatment, regular physical activity, car driver, having enough sleep (0 = yes, 1 = no), use of dental health checks and having any oral health problems (0 = no, 1 = yes), and elevation (0 = ≤258 m, 1 = >258 m). Note that 0 as the reference category. OR: odds ratio; 95% CI: 95% confidence interval.
